# Intraperitoneal administration of tumor-targeting *Salmonella typhimurium* A1-R inhibits disseminated human ovarian cancer and extends survival in nude mice

**DOI:** 10.18632/oncotarget.3607

**Published:** 2015-03-16

**Authors:** Yasunori Matsumoto, Shinji Miwa, Yong Zhang, Ming Zhao, Shuya Yano, Fuminari Uehara, Mako Yamamoto, Yukihiko Hiroshima, Makoto Toneri, Michael Bouvet, Hisahiro Matsubara, Hiroyuki Tsuchiya, Robert M. Hoffman

**Affiliations:** ^1^ AntiCancer, Inc, San Diego, CA, USA; ^2^ Department of Surgery, University of California San Diego, San Diego, CA, USA; ^3^ Department of Frontier Surgery, Graduate School of Medicine, Chiba University, Chiba, Japan; ^4^ Department of Orthopaedic Surgery, Graduate School of Medical Sciences, Kanazawa University, Ishikawa, Japan

**Keywords:** ovarian cancer, orthotopic, mouse model, bacterial therapy, Salmonella typhimurium A1-R

## Abstract

Peritoneal disseminated cancer is highly treatment resistant. We here report the efficacy of intraperitoneal (i.p.) administration of tumor-targeting *Salmonella typhimurium* A1-R in a nude mouse model of disseminated human ovarian cancer. The mouse model was established by intraperitoneal injection of the human ovarian cancer cell line SKOV3-GFP. Seven days after implantation, mice were treated with *S. typhimurium* A1-R via intravenous (i.v.) or i.p. administration at the same dose, 5×10^7^ CFU, once per week. Both i.v. and i.p. treatments effected prolonged survival compared with the untreated control group (P=0.025 and P<0.001, respectively). However, i.p. treatment was less toxic than i.v. treatment. Tumor-specific targeting of *S. typhimurium* A1-R was confirmed with bacterial culture from tumors and various organs and tumor or organ colony formation after i.v. or i.p. injection. Selective tumor targeting was most effective with i.p. administration. The results of the present study show *S. typhimurium* A1-R has promising clinical potential for disseminated ovarian cancer, especially via i.p. administration.

## INTRODUCTION

Ovarian cancer is a leading cause of cancer-related death among women in the U.S. with 22,000 being diagnosed and 14,000 deaths each year [1]. If the cancer has distantly spread from the ovary, the 5-year survival rate is 27% [2, 3] since platinum-based chemotherapy, which is first-line, for ovarian cancer with peritoneal dissemination [4], has low efficacy.

For more than 200 years cancers have been observed to regress following acute bacterial infections, mostly streptococcal [5]. In the late 19th and early 20th centuries. Coley infected cancer patients with *Streptococcus pyrogenes* and later treated the patients with extracts of the bacteria, which became known as Coley's toxins. Coley often had very good results with both the bacteria and the toxins [6].

Recently, there has been intense interest to develop bacterial therapy of cancer [6, 7]. The barriers in tumors for standard therapy such as hypoxia, acidic pH, disorganized vascular architecture, are beneficial for bacteria to target cancer [7].

One approach to bacterial therapy of cancer is to use anaerobic bacteria such as *Bifidobacterium* [8] and *Clostridium* [9] which replicate in necrotic areas of tumors. Anaerobic bacteria cannot grow in oxic viable tumor tissue, which restricts their efficacy. In addition, obligate anaerobic bacteria may be limited to intratumor (i.t.) injection which would preclude their use for metastatic cancer.

Recently a human patient with metastatic leiomyosarcoma was treated by i.t. injection of *Clostridium novyi* (*C. novyi*)-NT spores which reduced the tumor within and surrounding the bone [10].

*Salmonella typhimurium*, which is a facultative anaerobe, was previously attenuated with purine and other auxotrophic mutations, as well as lipid A-modified (msbB), and termed VNP20009 [11], VNP20009 was tested in a Phase I clinical trial on patients with metastatic melanoma and renal cell carcinoma [12].

Another strain of *S. typhimurium*, A1-R, has been developed by our laboratory and has increased antitumor efficacy compared to VNP20009 [13]. *S. typhimurium* A1-R is auxotrophic for Leu-Arg which prevents it from mounting a continuous infection in normal tissues. A1-R has no other attenuating mutations as does VNP20009 and, therefore, has higher tumor virulence. A1-R was able to eradicate primary and metastatic tumors in monotherapy in nude mouse models of prostate, breast, lung and pancreatic cancer, as well as sarcoma and glioma [14-22]. Tumors with a high degree of vascularity were more sensitive to A1-R, and vascular destruction appears to play a role in A1-R antitumor efficacy [23, 24].

*S. typhimurium* A1-R can target chemo-resistant pancreatic cancer stem-like cells [25] and pancreatic cancer patient-derived PDOX orthotopic xenographs [26].

In a recent study, we demonstrate that A1-R inhibits tumor growth, dissemination, and metastasis and extends survival in mouse models of aggressive human ovarian cancer [27].

The present study demonstrates *S. typhimurium* A1-R is highly effective for disseminated ovarian cancer, especially when administered i.p.

## RESULTS AND DISCUSSION

### *S. typhimurium* A1-R inhibits ovarian cancer cell proliferation *in vitro* in a dose-dependent manner

**Figure 1:**
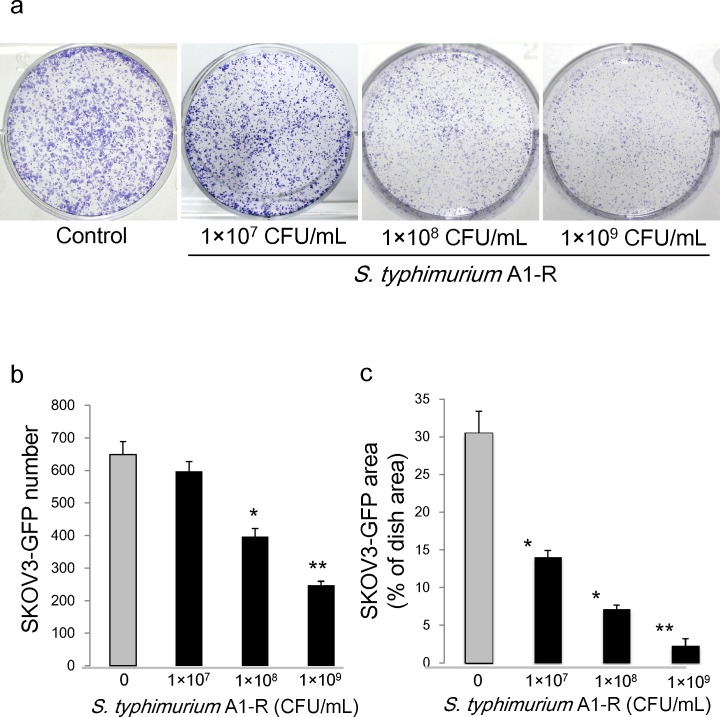
Efficacy of *S.typhimurium* A1-R on ovarian cancer cells *in vitro* a. Clonogenic assays were performed. b. SKOV3-GFP colony number after *S. typhimurium* A1-R treatment. c. SKOV3-GFP colony area after *S. typhimurium* A1-R treatment. *P < 0.05, **P < 0.01 compared with the control group.

*S. typhimurium* A1-R was effective on SKOV3-GFP ovarian cancer cells *in vitro*. The number of SKOV3-GFP colonies was 649.3 ± 39.6 in the control group; 597.3 ± 30.4 when treated with 1×10^7^ CFU/ml *S. typhimurium* A1-R; 396.7 ± 25.0 when treated with 1×10^8^ CFU/ml *S. typhimurium* A1-R; and 247.0 ± 12.7 when treated with 1×10^9^ CFU/ml of *S. typhimurium* A1-R (Fig. 1a,b). SKOV3-GFP colony areas were 30.5 ± 2.9(% of dish area) in control group; 14.1 ± 0.8(% of dish area) when treated with 1×10^7^ CFU/ml of *S. typhimurium* A1-R; 7.17 ± 0.5(% of dish area) when treated with 1×10^8^ CFU/ml of *S. typhimurium* A1-R; and 2.30 ± 0.9(% of dish area) when treated with 1×10^9^ CFU/ml of *S. typhimurium* A1-R (Fig. 1c). These results indicate that *S. typhimurium* A1-R inhibits proliferation of SKOV3-GFP cells in a dose- dependent manner.

### Establishment of the nude-mouse model of human ovarian cancer peritoneal dissemination

**Figure 2:**
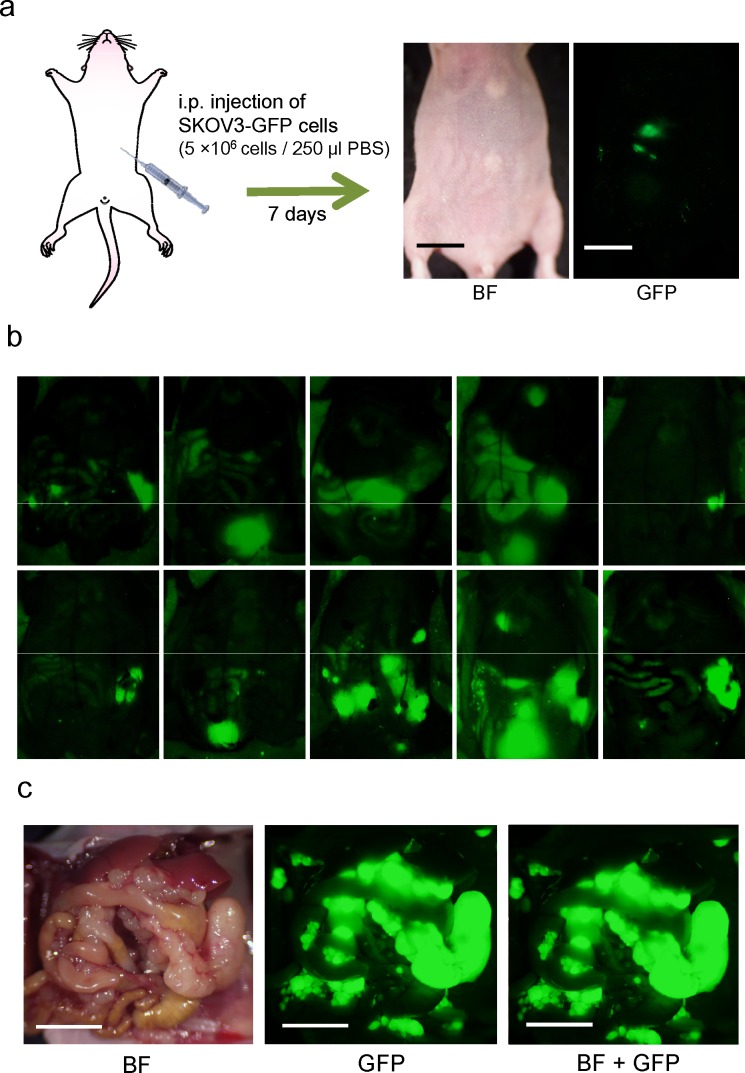
Nude mouse model of disseminated ovarian cancer a. Within seven days after i.p. injection of SKOV3-GFP cells (5×10^6^ cells in 250 μl PBS), disseminated tumors appeared, visualized by fluorescence imaging (Bar: 1 cm). b. Fluorescence imaging via the peritoneum on day 14. c. Representative intraperitoneal imaging at death. (Bar: 1 cm) BF: bright field.

Two weeks after i.p. implantation of SKOV3-GFP cells (5×10^6^), tumor dissemination was confirmed in all 10 mice on the peritoneum visualized with GFP imaging (Fig. 2). Tumors were confirmed at sacrifice and laparotomy. Disseminated tumors growing in the abdominal cavity led to animal death at approximately 40 days (Fig. 2).

### *S. typhimurium* A1-R therapy extended the survival period of the peritoneal dissemination model

**Figure 3:**
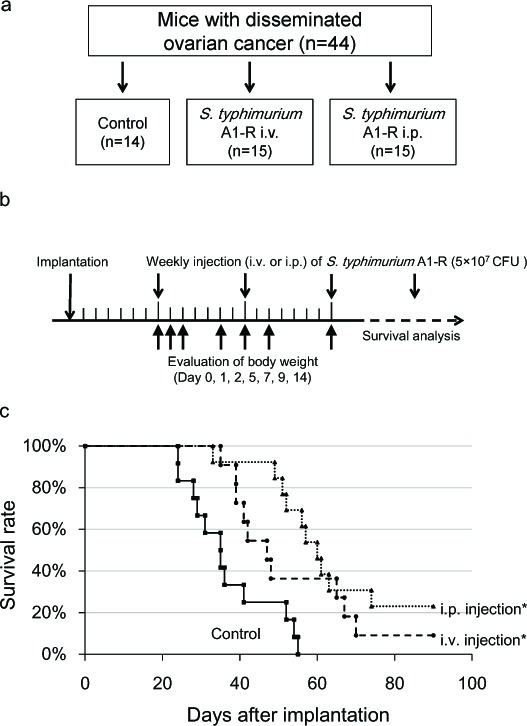

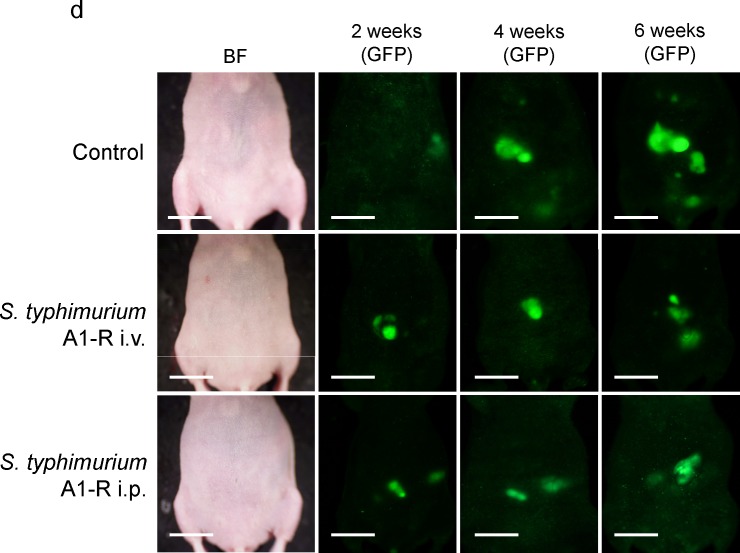
*S. typhimurium* A1-R treatment of disseminated ovarian cancer a. Study design for comparing the route of administration of *S. typhimurium* A1-R. b. Treatment schedule. On day 0, SKOV3-GFP cells were (5×10^6^) injected into the peritoneal cavity in 45 nude mice. On day 7, tumor formation was confirmed in 44 mice. Mice were divided into three groups. *S. typhimurium* A1-R (5×10^7^ CFU) was injected i.v. or i.p. once a week starting from day 7. To assess the toxicity of A1-R treatment, the body weight of all mice was measured on days 0, 1, 2, 5, 7, 9, 14. c. Survival curves of treated and control groups of mice. The survival period of the treated mice was significantly prolonged compared with the untreated control group (P = 0.025 in i.v. group; P < 0.001 in i.p. group). d. Representative time-course images of treated and control mice (bright-field [BF] and GFP imaging, Bar: 1 cm)

Nude mice with disseminated ovarian cancer were divided into three groups: untreated control; treatment with i.v. injection of *S. typhimurium* A1-R (5×10^7^ CFU); and treatment with i.p. injection of *S. typhimurium* A1-R (5×10^7^ CFU). Median survival in the control group was 35 days; i.v.- treated group, 47 days; and i.p.- treated group 60 days. One mouse in the i.v.-treated group and three mice in the i.p. -treated group survived to day 90. Treatment with i.v. or i.p. injection of *S. typhimurium* A1-R significantly prolonged the survival period compared with the control group (*p* = 0.025 and *p* < 0.001, respectively, Fig. 3c, d).

The body weight was compared among the three groups to assess the toxicity of bacterial therapy. Maximum body weight loss was 8.8% in the i.v. group on day 1, and 5.8% in the i.p group on day 2. The body weight recovered by day 5 in the i.p. group. These results indicate that i.p. bacterial therapy is more effective for disseminated ovarian cancer and less toxic than i.v. injection.

### *S. typhimurium* A1-R administrated i.p. is eliminated from normal tissues

**Figure 4:**
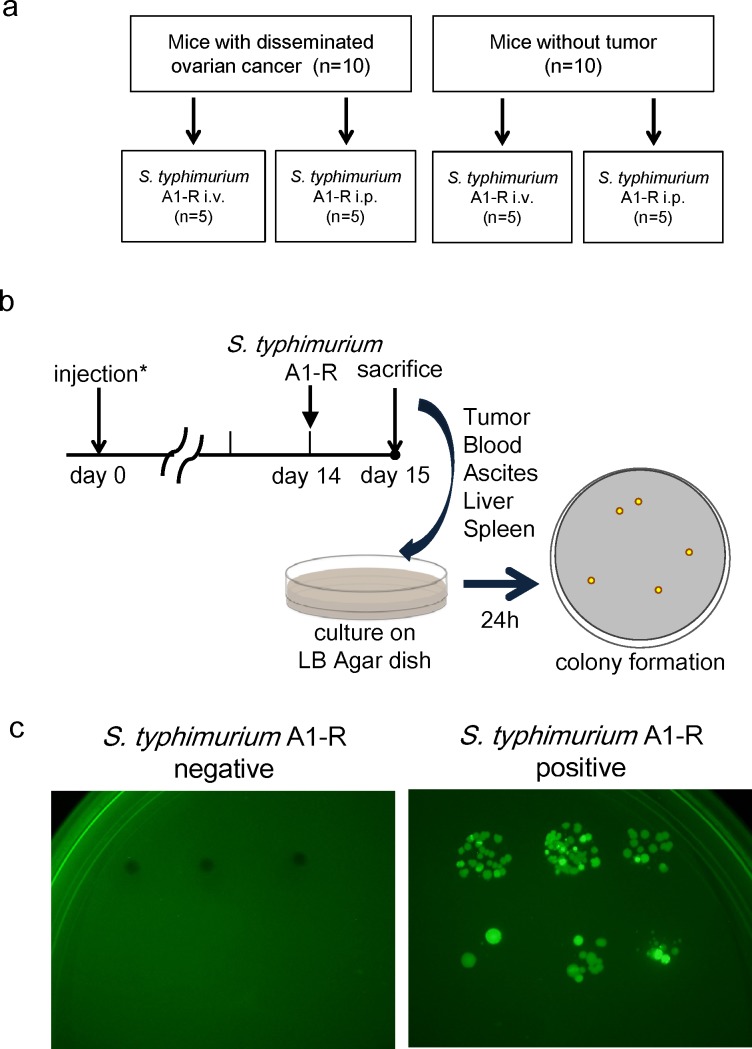
Selective tumor-targeting of *. typhimurium* A1-R after different routes of treatment a. Study design. Twenty mice were divided into two groups: injection of SKOV3-GFP cells (5×10^6^ cells) or PBS into the peritoneal cavity. Half of the mice in each group were treated with i.v. or i.p. injection of *S. typhimurium* A1-R. b. Schedule of the study. Fourteen days after injection, *S. typhimurium* A1-R (5 × 10^7^ CFU) were injected i.v. or i.p. into each of five mice with or without tumor. Twenty-four hours after treatment, all mice were sacrificed. Blood, ascites, liver, spleen, and tumors were harvested for culture of *S. typhimurium* A1-R. Each tissue was seeded on LB-Agar medium with serial dilution in triplicate. Colony formation was assessed after 24 hours with fluorescence imaging (OV-100). c. Representative negative and positive findings of *S. typhimurium* A1-R colony formation

Ten nude mice without tumor were also treated by i.v. or i.p. injection of *S. typhimurium* A1-R (Fig. 4a). Twenty-four hours after bacterial injection, blood, ascites, liver, spleen and tumor were harvested from each mouse and seeded on LB-Agar dishes. After 24-hour culture, *S. typhimurium* A1-R colony formation was assessed with fluorescence imaging (OV100, Olympus, Japan) (Fig. 4b, c). In mice without tumors, no *S. typhimurium* A1-R was detected when treated i.p. In contrast, spleens in two mice treated with i.v. injection of *S. typhimurium* A1-R had colony formation. In mice with disseminated ovarian cancer, i.p. injection resulted in higher amounts of *S. typhimurium* A1-R in the tumors. In contrast, i.v. injection of *S. typhimurium* resulted in bacterial colonies grown from blood, liver, and spleen as well as tumor (Table 1). This result indicates that *S. typhimurium* A1-R can be eliminated from tissues in mice without tumors and *S. typhimurium* A1-R administrated i.p. had greater tumor specificity then when administered i.v.

Our results demonstrate that i.p. administration of *S. typhimurium* A1-R has higher antitumor efficacy and less toxicity in nude mouse models of disseminated ovarian cancer, demonstrating clinical potential for this currently highly treatment-resistant disease.

The tumor-targeting of disseminated ovarian cancer strategy developed in the present report could also be used with previously-developed tumor targeting strategies [28-35].

**Table 1:** *S. typhimurium* A1-R colony formation on LB agar

	Tumor (−)		Tumor (+)	
	ip	iv	ip	iv
Tumor	N/A	N/A	5/5	3/5
Blood	0/5	0/5	0/5	1/5
Ascites	0/5	0/5	2/5	0/5
Liver	0/5	0/5	1/5	5/5
Spleen	0/5	2/5	2/5	4/5

N/A: Not available

## MATERIALS AND METHODS

### Cell line and culture conditions

The SKOV3-GFP [36] human ovarian cancer cell line (AntiCancer, Inc., San Diego, CA) was used for this study. Cells were maintained in Dulbecco's modified Eagle's medium supplemented with 10% fetal bovine serum (Sigma-Aldrich). All media were supplemented with penicillin and streptomycin (Gibco-BRL). Cells were cultured at 37°C with 95% air and 5% CO_2_.

### Mice

Athymic nude mice (nu/nu) (AntiCancer, Inc.), 7 weeks, were used. Mice were kept in a barrier facility under HEPA filtration. Mice were fed with autoclaved laboratory rodent diet. All animal studies were conducted in accordance with the principles and procedures outlined in the NIH guide for the Care and Use of Laboratory Animals under assurance number A3873-1.

### Preparation of *S. typhimurium* A1-R for treatment

GFP-expressing *S. typhimurium* A1-R bacteria (AntiCancer Inc., San Diego, CA, USA) were grown overnight in LB medium and then diluted 1:10 in LB medium. Bacteria were harvested at late-log phase, washed with PBS, and then diluted in PBS. Bacteria were then ready for injection in mice [16].

### Clonogenic assay

SKOV3-GFP cells were grown in 6-well plates to a density of approximately 1×10^3^ cells/well. *S. typhimurium* A1-R GFP were diluted in cell culture medium and added to the cancer cells at either 1×10^7^, 1×10^8^ or 1×10^9^ CFU/ml and incubated for 60 min at 37°C. The cells were rinsed and cultured in medium containing gentamycin sulfate (20 μg/ml) to kill external, but not internal, bacteria. After seven days culture, cancer-cell colonies were fixed with ethanol and then stained with crystal violet. The number of colonies and the ratio of colony areas were assessed by ImageJ (National Institutes of Health, Bethesda, MD).

### Dissemination model of ovarian cancer in nude mice

To establish a mouse model of ovarian cancer dissemination in the peritoneal cavity, SKOV3-GFP cells (5×10^6^) suspended in 250 μl PBS were injected into the peritoneal cavity in nude mice. Two weeks after implantation, tumor formation were assessed with fluorescence imaging and laparotomy. The Olympus OV100 Small Animal Imaging System (Olympus, Tokyo, Japan) was used for whole body imaging in live mice [37].

### *S. typhimurium* A1-R therapy of disseminated ovarian cancer in nude mice

Seven days after the implantation of SKOV3-GFP cells as described above, intra-abdominal tumor formation was confirmed with fluorescence imaging in 44 mice. The 44 mice were divided into three groups (Fig. 3a):

1) Treatment with i.v. injection of *S. typhimurium* A1-R (n = 15).

*S. typhimurium* A1-R (5×10^7^ CFU) in 100 μl PBS was injected i.v. once every seven days starting seven days post-implantation.

2) Treatment with i.p. injection of *S. typhimurium* A1-R (n = 15).

*S. typhimurium* A1-R (5×10^7^ CFU) in 100 μl PBS was injected i.p. once every seven days, starting seven days after cell injection.

3) No treatment group (n = 14).

The overall survival time of each group was determined. The body weight of all mice was measured at day 0, 1, 2, 5, 7, 9, 14 in order to assess toxicity of *S. typhimurium* A1-R therapy.

### Tumor selectivity of *S. typhimurium* A1-R

SKOV3-GFP (5×10^6^ cells) were injected i.p. in 10 nude mice. Fourteen days after the implantation, five mice were treated with i.v. injection of *S. typhimurium* A1-R (5×10^7^ CFU) and another five mice were treated with i.p. injection of *S. typhimurium* A1-R (5×10^7^ CFU). Ten nude mice without tumor were also treated with *S. typhimurium* A1-R i.v. or i.p., five mice each (Fig. 4a). Twenty-four hours after bacterial administration, blood, ascites, liver, spleen and tumor were harvested and seeded in LB-Agar dishes. After 24-hour culture, *S. typhimurium* A1-R colony formation was assessed with fluorescence imaging (OV100, Olympus, Japan) (Fig. 4b, c).

### Statistical analysis

Data comparisons between two groups were assessed using the Student's *t*-test. When more than two groups were assessed, analysis of variance (ANOVA) was used. The Kaplan-Meier method was used for survival analysis. The log-rank test was used for statistical significance of the difference between the two groups. Differences were considered significant when P < 0.05. Data are expressed as mean ± standard error (SE). Statistical analyses were performed with EZR (Saitama Medical Center, Jichi Medical University).

## References

[1] Siegel R, Naishadham D, Jemal A (2013). Cancer statistics 2013. CA Cancer J Clin.

[2] Howlader N, Noone AM, Krapcho M, Garshell J, Neyman N, Altekruse SF, Kosary CL, Yu M, Ruhl J, Tatalovich Z, Cho H, Mariotto A, Lewis DR, Chen HS, Feuer EJ, Cronin KA SEER Cancer Statistics Review, 1975–2010.

[3] American Cancer Society (2013). http://www.cancer.org/.

[4] Cannistra SA, Bast RC, Berek JS (2003). Progress in the management of gynecologic cancer: Consensus summary statement. J Clin Oncol.

[5] Coley William http://en.wikipedia.org/wiki/William_Coley.

[6] Hoffman RM (2012). The preclinical discovery of bacterial therapy for the treatment of metastatic cancer with unique advantages. Expert Opin Drug Discov.

[7] Forbes NS (2010). Engineering the perfect (bacterial) cancer therapy. Nat Rev Cancer.

[8] Yazawa K, Fujimori M, Nakamura T, Sasaki T, Amano J, Kano Y, Taniguchi S (2001). Bifidobacterium longum as a delivery system for gene therapy of chemically induced rat mammary tumors. Breast Cancer Res Treat.

[9] Dang LH, Bettegowda C, Huso DL, Kinzler KW, Vogelstein B (2001). Combination bacteriolytic therapy for the treatment of experimental tumors. Proc Natl Acad Sci USA.

[10] Roberts NJ, Zhang L, Janku F, Collins A, Bai RY, Staedtke V, Rusk AW, Tung D, Miller M, Roix J, Khanna KV, Murthy R, Benjamin RS, Helgason T, Szvalb AD, Bird JE, Roy-Chowdhuri S, Zhang HH, Qiao Y, Karim B, McDaniel J, Elpiner A, Sahora A, Lachowicz J, Phillips B, Turner A, Klein MK, Post G, Diaz LA, Riggins GJ, Papadopoulos N, Kinzler KW, Vogelstein B, Bettegowda C, Huso DL, Varterasian M, Saha S, Zhou S (2014). Intratumoral injection of Clostridium novyi-NT spores induces antitumor responses. Sci Transl Med.

[11] Pawelek JM, Low KB, Bermudes D (2003). Bacteria as tumour-targeting vectors. Lancet Oncol.

[12] Toso JF, Gill VJ, Hwu P, Marincola FM, Restifo NP, Schwartzentruber DJ, Sherry RM, Topalian SL, Yang JC, Stock F, Freezer LJ, Morton KE, Seipp C, Haworth L, Mavroukakis S, White D, MacDonald S, Mao J, Sznol M, Rosenberg SA (2002). Phase I study of the intravenous administration of attenuated *Salmonella typhimurium* to patients with metastatic melanoma. J Clin Oncol.

[13] Zhang Y, Zhang N, Hoffman RM, Zhao M Comparision of the selective targeting efficacy of *S. typhimurium* A1-R and VNP20009 on the lewis lung carcinoma in nude mice. Oncotarget 2015:Epub ahead of print.

[14] Zhao M, Yang Y, Li XM, Jiang P, Baranov E, Li S, Xu M, Penman S, Hoffman RM (2005). Tumor-targeting bacterial therapy with amino acid auxotrophs of GFP-expressing *Salmonella typhimurium*. Proc Natl Acad Sci USA.

[15] Zhao M, Yang M, Ma H, Li X, Tan X, Li S, Yang Z, Hoffman RM (2006). Targeted therapy with a Salmonella typhimurium leucine-arginine auxotroph cures orthotopic human breast tumors in nude mice. Cancer Res.

[16] Zhao M, Geller J, Ma H, Yang M, Penman S, Hoffman RM (2007). Monotherapy with a tumor-targeting mutant of *Salmonella typhimurium* cures orthotopic metastatic mouse models of human prostate cancer. Proc Natl Acad Sci USA.

[17] Hayashi K, Zhao M, Yamauchi K, Yamamoto N, Tsuchiya H, Tomita K, Kishimoto H, Bouvet M, Hoffman RM (2009). Systemic targeting of primary bone tumor and lung metastasis of high-grade osteosarcoma in nude mice with a tumor-selective strain of *Salmonella typhimurium*. Cell Cycle.

[18] Nagakura C, Hayashi K, Zhao M, Yamauchi K, Yamamoto N, Tsuchiya H, Tomita K, Bouvet M, Hoffman RM (2009). Efficacy of a genetically-modified *Salmonella typhimurium*in an orthotopic human pancreatic cancer in nude mice. Anticancer Res.

[19] Kimura H, Zhang L, Zhao M, Hayashi K, Tsuchiya H, Tomita K, Bouvet M, Wessels J, Hoffman RM (2010). Targeted therapy of spinal cord glioma with a genetically-modified *Salmonella typhimurium*. Cell Prolif.

[20] Yam C, Zhao M, Hayashi K, Ma H, Kishimoto H, McElroy M, Bouvet M, Hoffman RM (2010). Monotherapy with a tumor-targeting mutant of *Salmonella typhimurium* controls liver metastasis in a mouse model of pancreatic cancer. J Surg Res.

[21] Momiyama M, Zhao M, Kimura H, Tran B, Chishima T, Bouvet M, Endo I, Hoffman RM (2012). Inhibition and eradication of human glioma with tumor targeting *Salmonella typhimurium* in an orthotopic nude-mouse model. Cell Cycle.

[22] Uchugonova A, Zhao M, Zhang Y, Weinigel M, König K, Hoffman RM (2012). Cancer-cell killing by engineered Salmonella imaged by multiphoton tomography in live mice. Anticancer Res.

[23] Leschner S, Westphal K, Dietrich N, Viegas N, Jablonska J, Lyszkiewicz M, Lienenklaus S, Falk W, Gekara N, Loessner H, Weiss S (2009). Tumor invasion of Salmonella enterica serovar Typhimurium is accompanied by strong hemorrhage promoted by TNFa. PLoS ONE.

[24] Liu F, Zhang L, Hoffman RM, Zhao M (2010). Vessel destruction by tumor targeting *Salmonella typhimurium* A1-R is enhanced by high tumor vascularity. Cell Cycle.

[25] Hiroshima Y, Zhao M, Zhang Y, Maawy A, Hassanein MK, Uehara F, Miwa S, Yano S, Momiyama M, Suetsugu A, Chishima T, Tanaka K, Bouvet M, Endo I, Hoffman RM (2013). Comparison of efficacy of *Salmonella typhimurium* A1-R and chemotherapy on stem-like and non-stem human pancreatic cancer cells. Cell Cycle.

[26] Hiroshima Y, Zhao M, Maawy A, Zhang Y, Katz MH, Fleming JB, Uehara F, Miwa S, Yano S, Momiyama M, Suetsugu A, Chishima T, Tanaka K, Bouvet M, Endo I, Hoffman RM (2014). Efficacy of *Salmonella typhimurium* A1-R versus chemotherapy on a pancreatic cancer patient-derived orthotopic xenograft (PDOX). J Cell Biochem.

[27] Matsumoto Y, Miwa S, Zhang Y, Hiroshima Y, Yano S, Uehara F, Yamamoto M, Toneri M, Bouvet M, Matsubara H, Hoffman RM, Zhao M (2014). Efficacy of tumor-targeting *Salmonella typhimurium* A1-R on nude mouse models of metastatic and disseminated human ovarian Cancer. J. Cell. Biochem.

[28] Blagosklonny MV (2005). How cancer could be cured by 2015. Cell Cycle.

[29] Blagosklonny MV (2003). Tissue-selective therapy of cancer. Br J Cancer.

[30] Blagosklonny MV (2003). Matching targets for selective cancer therapy. Drug Discov Today.

[31] Blagosklonny MV (2008). “Targeting the absence” and therapeutic engineering for cancer therapy. Cell Cycle.

[32] Blagosklonny MV (2005). Teratogens as anti-cancer drugs. Cell Cycle.

[33] Blagosklonny MV (2001). Treatment with inhibitors of caspases, that are substrates of drug transporters, selectively permits chemotherapy-induced apoptosis in multidrug-resistant cells but protects normal cells. Leukemia.

[34] Blagosklonny MV (2006). Target for cancer therapy: proliferating cells or stem cells. Leukemia.

[35] Blagosklonny MV (2007). Cancer stem cell and cancer stemloids: from biology to therapy. Cancer Biol Ther.

[36] Buick RN, Pullano R, Trent JM (1985). Comparative properties of five human ovarian adenocarcinoma cell lines. Cancer Res.

[37] Yamauchi K, Yang M, Jiang P, Xu M, Yamamoto N, Tsuchiya H, Tomita K, Moossa AR, Bouvet M, Hoffman RM (2006). Development of real-time subcellular dynamic multicolor imaging of cancer-cell trafficking in live mice with a variable-magnification whole-mouse imaging system. Cancer Res.

